# Crocodiles Alter Skin Color in Response to Environmental Color Conditions

**DOI:** 10.1038/s41598-018-24579-6

**Published:** 2018-04-18

**Authors:** Mark Merchant, Amber Hale, Jen Brueggen, Curt Harbsmeier, Colette Adams

**Affiliations:** 1 Department of Chemistry and Physics, McNeese State University, Lake Charles, Louisiana USA; 2 Department of Biology, McNeese State University, Lake Charles, Louisiana USA; 3 999 Anastasia Blvd, St. Augustine, Florida USA; 4 Tampa’s Lowry Park Zoo, Tampa, Florida USA; 5 Gladys Porter Zoo, Brownsville, Texas USA

## Abstract

Many species alter skin color to varying degrees and by different mechanisms. Here, we show that some crocodylians modify skin coloration in response to changing light and environmental conditions. Within the Family, Crocodylidae, all members of the genus *Crocodylus* lightened substantially when transitioned from dark enclosure to white enclosures, whereas *Mecistops* and *Osteolaemus* showed little/no change. The two members of the Family Gavialidae showed an opposite response, lightening under darker conditions, while all member of the Family Alligatoridae showed no changes. Observed color changes were rapid and reversible, occurring within 60–90 minutes. The response is visually-mediated and modulated by serum α-melanocyte-stimulating hormone (α-MSH), resulting in redistribution of melanosomes within melanophores. Injection of crocodiles with α-MSH caused the skin to lighten. These results represent a novel description of color change in crocodylians, and have important phylogenetic implications. The data support the inclusion of the Malayan gharial in the Family Gavialidae, and the shift of the African slender-snouted crocodile from the genus *Crocodylus* to the monophyletic genus *Mecistops*.

## Introduction

The rapid alteration of skin color is well known among a wide assortment of ectothermic vertebrates and invertebrates^1^. Adaptive skin color changes may occur for a variety of reasons, including communication, thermal regulation, and crypsis^1^. The modification of skin pigmentation is achieved by either physiological or morphological mechanisms^1^. Physiological color change is typically influenced by changes in circulating hormone levels, that is in turn, controlled by neurological stimuli^2^, and the molecular mechanisms have been studied in detail^3^. Morphological color change, described in teleost fish^4^, amphibians^5^, and reptiles^6^, is generally slower, and involves changes in both the density and morphology of melanophores^7^.

Color change for the purposes of communication, as described by Korzan *et al*.^8^, may signal dominance, aggression, or reproductive state^9,10^. Color change in ectothermic vertebrates may also function to enhance thermoregulatory ability^11^, a phenomenon that has been documented in lizards^12–14^ and toads^15^. The change of skin color to match environment is typically used for camouflage to either avoid predation or aid in foraging success^1^. Numerous ectotherms across a broad spectrum of taxa^6,16^ including invertebrates (crustaceans and cephalopods)^17–19^, and vertebrates (fishes, amphibians, and reptiles)^20–22^ utilize color change for crypsis. Rapid and complex alterations in skin color have been well-documented in cuttlefish, squid, octopuses^23^ and insects^1^. In addition, several classes of vertebrates have been shown to adapt body shading in response to environmental color changes. Chameleons^1^, anoles^24^, frogs^25–29^ and fish^21^ alter skin color in response to environmental changes.

Crocodylians were first reported to alter skin colors with respect to their backgrounds in 1985^30^. Saltwater crocodile (*Crocodylus porosus*) hatchlings developed a dark or light color when raised in dark or light tanks, respectively, for three months. These same animals reverted to the opposite color three weeks after being placed in a tank of opposite color. Since crocodylian hatchlings and small juveniles typically experience high rates of predation in the wild, and only a small percentage of young survive to adulthood^31,32^, the ability to adapt to different environments may be a key to avoid predation. In addition, since crocodylians are slow, methodic, stalking hunters, the ability to alter skin color to match surroundings would certainly aid in predation^33^.

Here we report the varying abilities of all 24 species of extant crocodylians to alter skin coloration in response to environmental light conditions, as well as the physiological mechanism that regulates color change in the genus *Crocodylus*. The ability to alter skin color, and type of alteration, is strongly divided along phylogenetic lines.

## Results and Discussion

**Figure 1:**
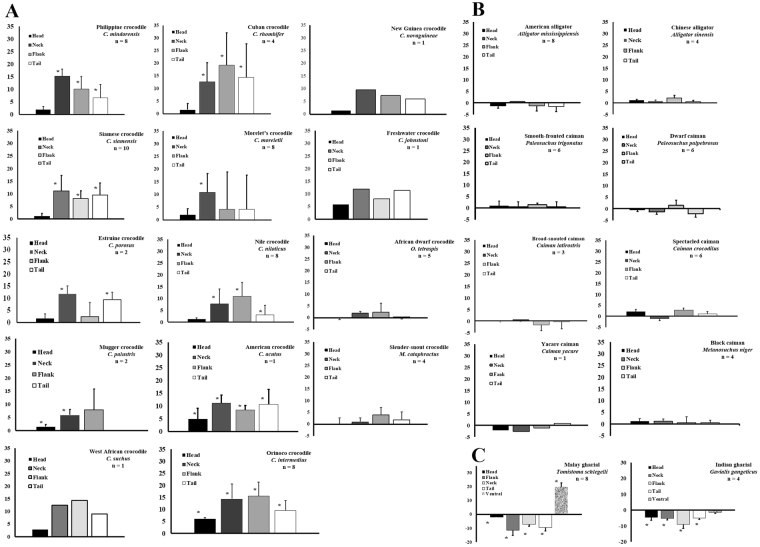
Effects of light environment on skin color of all 24 species of crocodylians. Animals maintained in black tanks were measured, placed in a white tank and measured again after three hrs. (**A**) Members of the Family Crocodylidae show relative strong color change, except for the African slender-snouted and African dwarf crocodiles. (**B**) Members of the Family Alligatoridae do not exhibit color change while members of the Family Gavialidae (**C**) change in the opposite manner to the crocodylids. The data represent the means ± standard deviations for the number of animals indicated on each graph. *Statistically different from measurements of skin color of the same animals in dark tanks.

**Figure 2:**
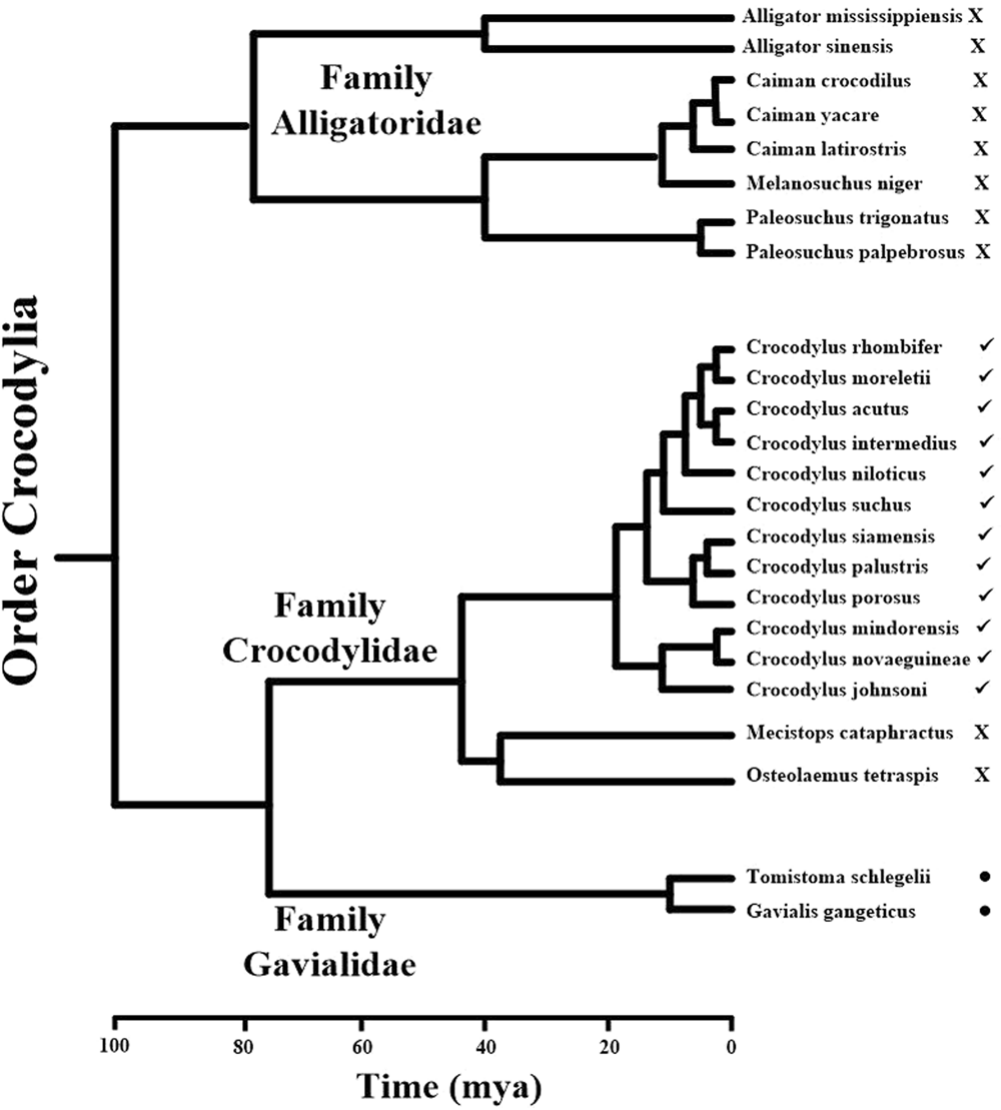
Phylogenetic relation of extant crocodylians, and their color change responses. The symbols adjacent to each species represent the following: **✓**Skin change to a lighter color in a light environment, **●**Skin change to a darker color in a lighter environment, X = no skin coloration change.

Most members of the Family Crocodylidae, and all members of the genus *Crocodylus*, exhibited alteration of skin color in response to background conditions (Fig. 1A). Crocodylids lighten the dorsolateral skin surfaces in light backgrounds and darken them in darker ones, but the ventral surface of crocodylids does not change color in response to environmental changes. In contrast, members of the Family Alligatoridae exhibit little or no ability to alter skin color in differing light conditions (Fig. 1B). These data suggest that development of the ability to change color with changing background conditions occurred after the split of these two families, approximately 80 million years ago^34^ (Fig. 2). The only two extant members of the Family Crocodylidae, which are not included in the genus *Crocodylus*, the African dwarf crocodile (*Osteolaemus tetraspis*) and the African slender-snouted crocodile (*Mecistops cataphractus*), showed little/no ability to change color. This implies that the common ancestor to the genus *Crocodylus* developed the capability to alter skin color after 30–40 mya (when the common ancestor for these two species split from the genus *Crocodylus*) and prior to 12–17 mya, when a rapid divergence of the genus *Crocodylus* occurred (Fig. 2). Alternatively, but a less likely scenario, is that the common ancestor to the Family Crocodylidae was able to change color, but the common ancestor to *M. cataphractus* and *O. tetraspsis* lost this ability after they diverged from the genus *Crocodylus*, but before the further divergence into separate species (Fig. 2).

Social interactions are known to influence skin coloration in some taxa of lizards^1,30^ and fish^35^. Since many crocodylian species live in communal populations, the chance for social interactions could potentially affect the results of color change studies. It is important to note that the color changes described in this study were induced in animals housed alone in individual tanks, and thus social interactions should not have influenced the results.

The two members of the Family Gavialidae, *Tomistoma schlegelii* and *Gavialis gangeticus* exhibited responses that were opposite of those demonstrated by members of the genus *Crocodylus*, yet similar in magnitude. Gharials responded to increased light with darkening of the dorsolateral skin surfaces (Fig. 1C). As mentioned, the ventral surface of the crocodylids did not change color, while juvenile Malaysian gharials respond to visible light with ventral darkening (Fig. 1C).

**Figure 3:**
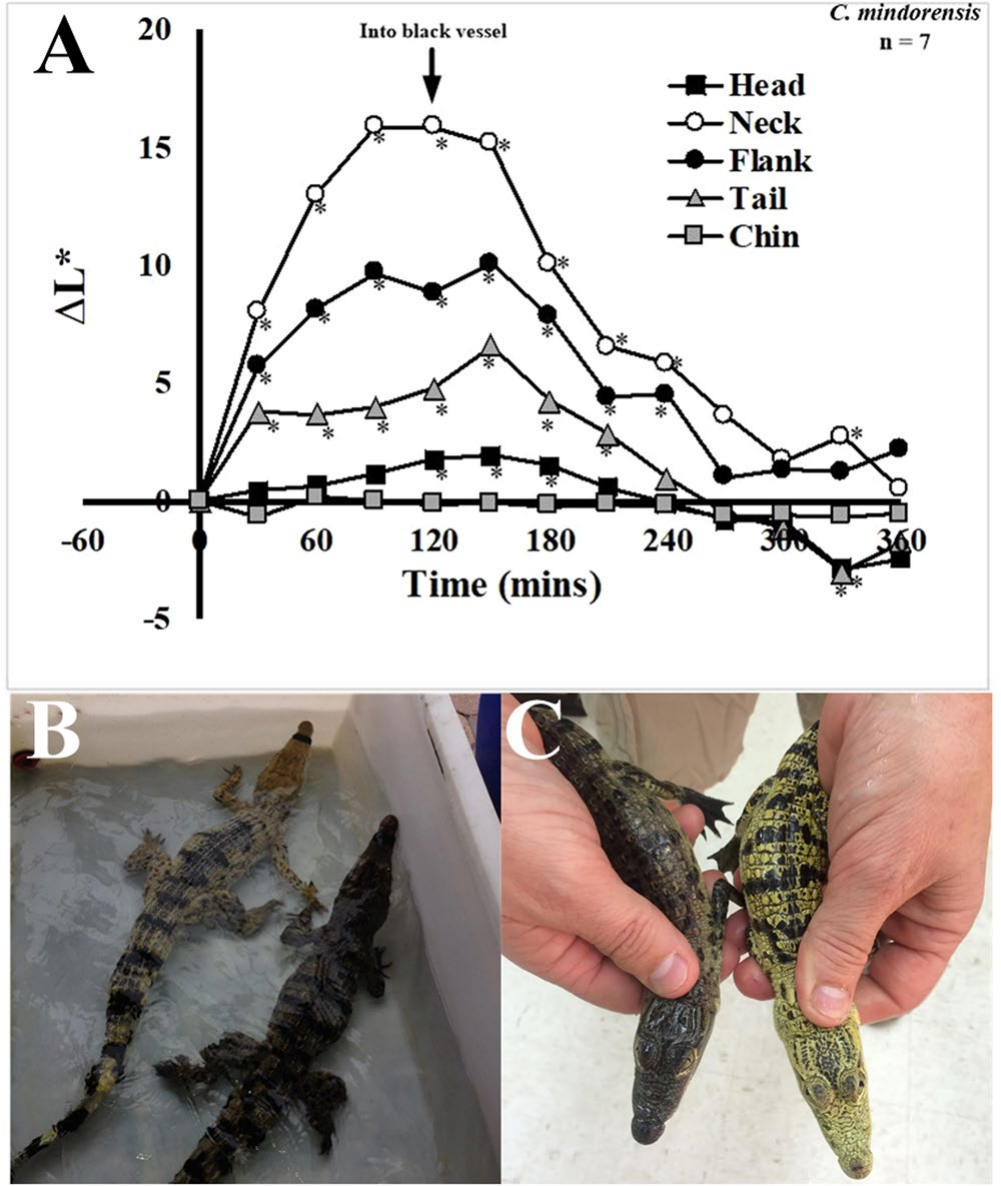
Magnitude of color change in crocodylians. (**A**) Analysis of dynamic color change in the Philippine crocodile. Animals acclimated to black tanks were placed in white environments and the color was measured every 30 min until stable. Animals were then placed back into black tanks and skin color was measured every 30 min until stable. *Significance from measurement at time zero. Color change of sibling Philippine (**B**) and Morelet’s (**C**) crocodiles after three hours in white tanks. The animals that appear darker in color were maintained in black tanks for three hours.

Maximum color change was achieved two hours after moving animals from white to dark colored enclosures, as demonstrated using Philippine crocodiles (*C. mindorensis*; Fig. 3A). After placement of the animals back into the white tank, the skin color reverted back to a lighter color. These changes occurred more quickly than the changes described by Kirshner^30^. The drastic alteration of skin color is visible to the unaided eye, as shown in pairs of sibling Philippine (Fig. 3B) and Morelet’s (*C. moreletii*, Fig. 3C) crocodiles, with one being held in a white tank for three hours and the other in a dark tank for the same time period. This relatively rapid change in skin color is indicative of a hormonally-controlled physiological response^2,3^.

**Figure 4:**
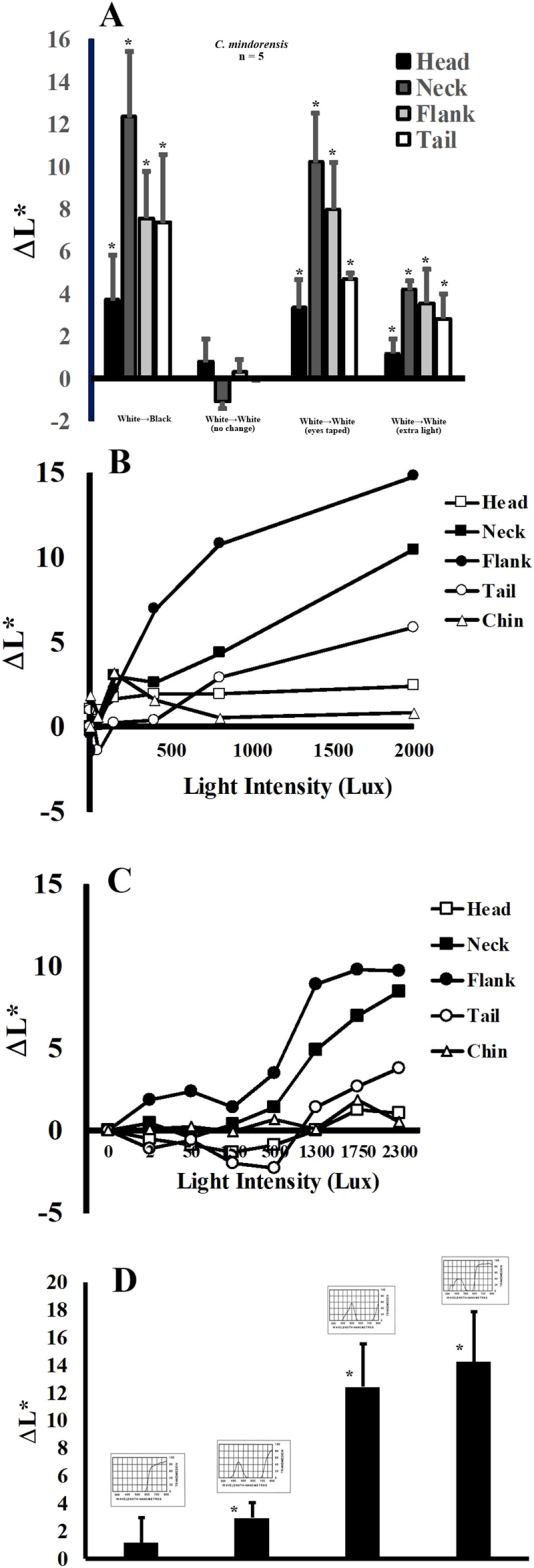
Characterization of crocodylian color change. (**A**) Philippine crocodiles in a white environment exhibit light coloration of their dorsolateral skin, and a darker color in a black environment. Covering the eyes resulted in skin darkening in a white environment. Placement of a lightened animal into a black environment flooded with extra light, results in minimal darkening of the skin. (**B**) Dependence of crocodile skin color change on light intensity. (**C**) The color change response is caused by light of shorter wavelengths in the visible spectrum (370–430 nm). *Statistically different from measurements of skin color of the same animals in dark tanks.

To determine if the mechanism by which light exposure influences skin color in crocodylids was physiological or morphological, the eyes of several Philippine crocodiles, maintained in light color environments, were taped such that no light could penetrate the eyes (Fig. 4A). Although the animals were placed back into their white environments, their skin darkened substantially within two hours. The change in skin color was similar to results obtained when the same animals were removed from white tanks and placed in black tanks without tape over their eyes. In addition, the movement of the crocodiles from white tanks to black tanks that were flooded with additional light resulted in only minimal darkening of the skin (Fig. 4A). Similarly, Philippine crocodiles placed in white tanks and maintained under low-light conditions exhibited dark skin tones. These results suggest that ocular stimulation with light plays a key role in the induction of color change. The black tanks absorb most of the light and reduce the amount of luminance reaching the animal’s eyes. Likewise, in a white tank, more indirect light is reflected toward the animals, resulting in more ocular stimulation and lighter skin color.

Keeble and Gamble^36^ proposed that the control of skin tone was dependent on albedo, the ratio of reflected light to direct light that reaches the eye. This implies that polarized light, which is more abundant in reflected light, might have a role in color change, and would also explain why flooding a black tank with higher intensity light does not cause the maximal color change effect (Fig. 4A), since the direct light is less effective and dark tank does not effectively reflect light to the animal.

Exposure to increased light intensity resulted in enhanced skin lightening in both Philippine and Morelet’s crocodiles (Fig. 4B). The results show that the Philippine crocodile was more sensitive to low light, and exhibited significant color changes at light intensities as low as 400 lux (neck and flank), while the tail and head responded with significant change at 800 lux (Fig. 4B). Similarly, the neck and flank regions of Morelet’s crocodiles exhibited color change at 500 lux, but the head and tail areas did not respond until 1750 lux. These results could be due to different amounts of melanophores in the skin of these crocodiles in different regions of the body. Alternatively, Philippine crocodiles might respond to lower light intensities with the production of higher levels of α-melanocyte stimulating hormone (αMSH). A combination of these and other factors is also possible.

Illumination of Morelet’s crocodiles with light of different spectral properties results in variable intensity of changes in skin color (Fig. 4C). We employed spectral filters to allow specific ranges of wavelengths to reach the animals. Exposure of these crocodiles to low energy light at the red end of the visible spectrum (500–700 nm, Fig. 4C) elicited minimal response. However, light of shorter wavelengths (350–450 nm) produced a much stronger response. Saltwater (*C. porosus*) and Australian freshwater (*C. johnsonii*) crocodiles have cone photoreceptors which absorb light at 424 and 426 nm, respectively^37^. Since the wavelength of light for these photoreceptors closely match the energy for light that stimulates this response, it is reasonable to expect that they may facilitate this physiological response.

Because crocodylians are ectothermic vertebrates, temperature is always a concern in experimental design, and adding light to an environment can increase temperature, thus influencing results. The results obtained from constant temperature experiments, during which only light was increased, suggest that the reported color change is not caused by changes in thermal environment (Fig. 4B). In addition, other mechanistic experiments, during which color changes were induced by simply taping the eyes (Fig. 4A), occurred at constant temperature. Due to experimental design, it is clear that changes in temperature are not responsible for these modifications in skin coloration.

**Figure 5:**
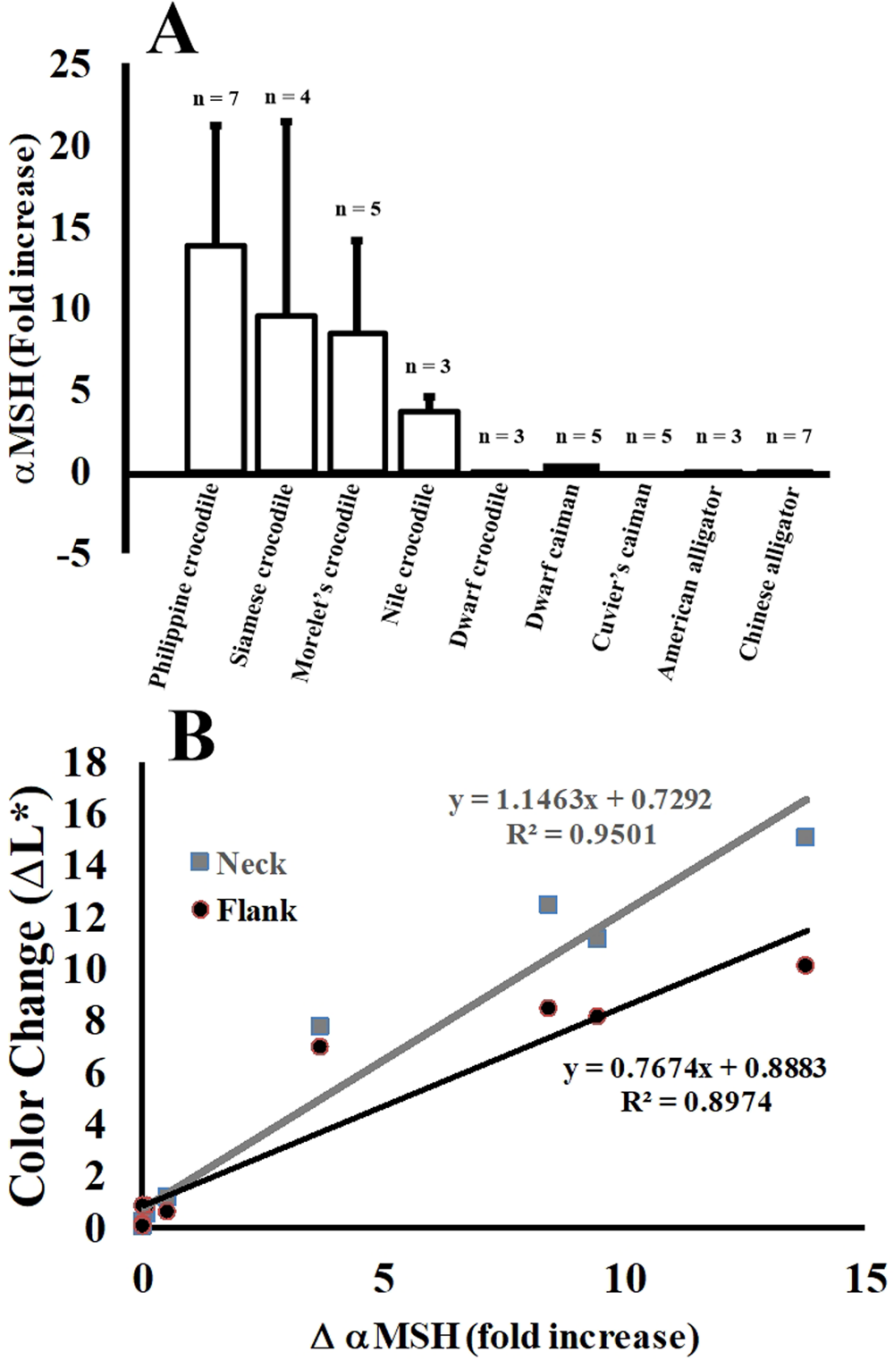
Crocodylian color change is mediated by αMSH and unaffected by corticosteroid. (**A**) Differential expression of αMSH in white and black environments. Blood was collected from animals maintained in black tanks, and then three hours later after acclimation to white tanks. *Statistically different from measurements of skin color of the same animals in dark tanks. (**B**) Correlation of changes in skin color with changes in αMSH levels.

Many vertebrates induce changes in skin color by increasing concentrations of circulating hormones. Changes in circulating αMSH have been linked to skin color changes in frogs^38^, lizards^2^, and fish^39^. The results in Fig. 5A show that members of the genus *Crocodylus* express αMSH at low levels when adapted to dark tanks, but at much higher concentrations when acclimated to lighter environments. Members of the Family Alligatoridae, which do not change color (Fig. 1B), also do not express differential levels of αMSH in response to environmental light changes (Fig. 5A). In crocodylids, the changes in circulating αMSH correlate well with the changes in both neck and flank skin color (Fig. 5B). There is a linear relationship between the change in the amount of plasma αMSH and the change in skin color. This provides strong evidence that the increased in expression of αMSH is linked to physiological changes in skin color in response to environmental light conditions.

**Figure 6:**
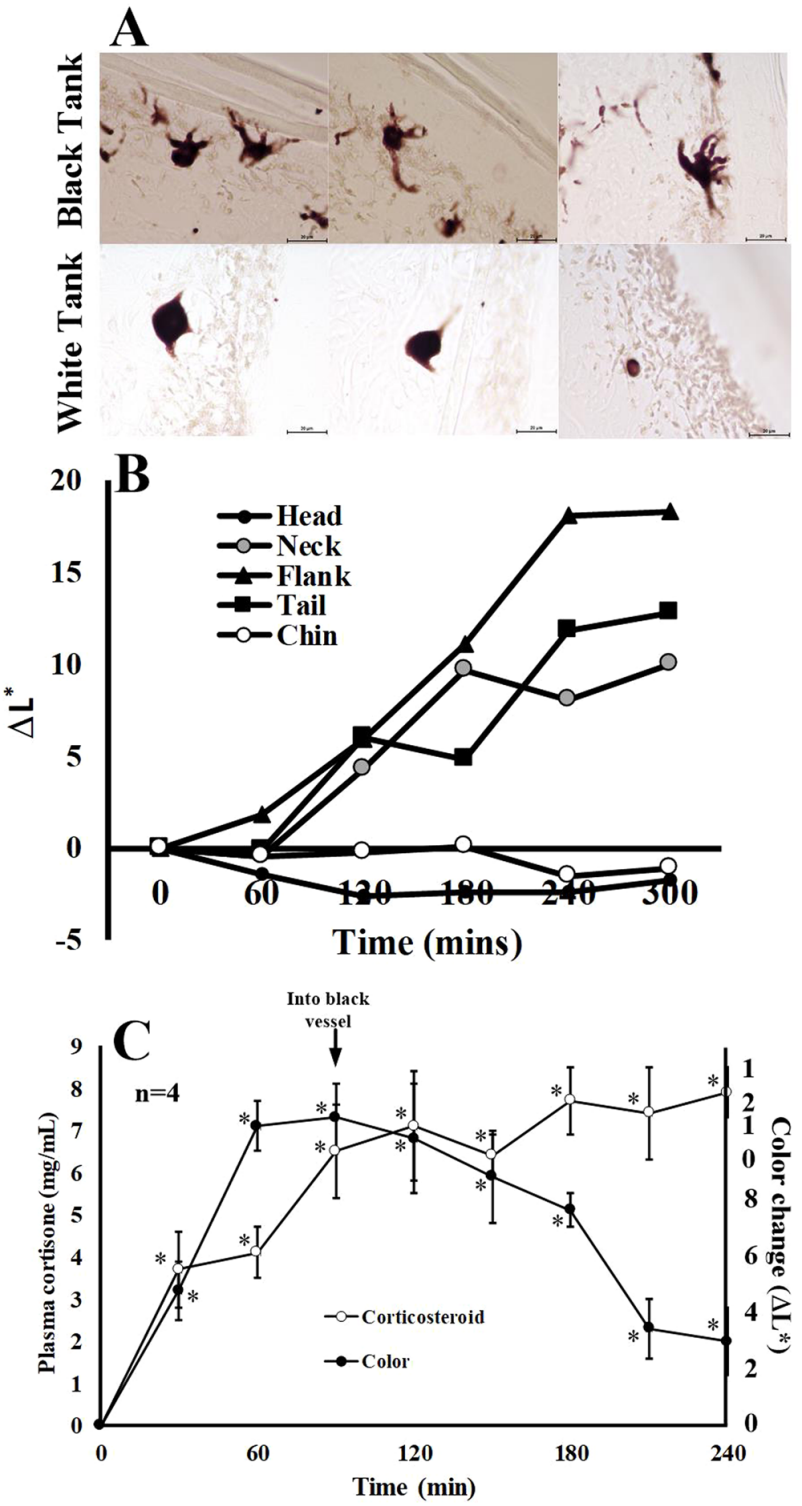
Effects of αMSH on crocodylian color change. (**A**) The top panels represent histological sections of skin from crocodiles maintained in dark tanks, while the bottom panels are skin samples from the same animals acclimated to white tanks for three hours. (**B**) Crocodiles acclimated to total darkness were injected with 10 μg/kg body αMSH. The animals were kept in darkness and skin color was measured for five hours. (**C**) Crocodiles maintained in black environments were moved to white tanks. A rapid rise in skin lightening was accompanied by a sharp increase in corticosteroid levels. When the crocodiles were placed back into a dark environment, skins color darkened while corticosteroid levels remained elevated. *Statistically different from measurements of skin color of the same animals in dark tanks.

The ultrastructure and cellular content of crocodylian skin was first described by Spearman and Riley^40^. Crocodylian skin contains a variety of pigment cells distributed throughout various layers^41^. The areas that are lighter in color exhibit sparse distributions of melanophores, while the black areas have two heavily-populated layers, one apical (epidermal) and another more basal (dermal). The data shown in Fig. 6A show that, in crocodiles conditioned to light tanks, the pigment organelles (melanosomes) inside the melanophores are condensed in the body of the cell. Under light conditions, when the melanosomes are concentrated in the body of the cell, the cells appear to be lighter in color and smaller, causing the skin to appear lighter. In contrast, when animals are acclimated to a dark environment, the pigment is distributed into the long cellular processes of the melanophores. Not only is the melanin expanded laterally, but is also spread upward toward the apical surface of the skin, making the yellow portions of the skin appear darker.

Stress-induced and corticosteroid-mediated interactions influence a broad spectrum of physiological and biochemical parameters in crocodylians^42,43^. Although stress from handling and restraint could have influenced the color changes observed in this study, the data suggest that the observed effects are not caused by stress. For instance, when the crocodylians were handled and restrained for this study, we measured increases in serum corticosteroid levels (Fig. 6C). When animals were placed in dark tanks and skin darkening was measured, corticosteroid levels were increased. However, when the animals were placed back in white tanks and the skins became lighter (Fig. 1A), the elevated corticosteroid levels persisted, and thus there was no correlation between steroid levels and color change. In other experiments (Fig. 3C), animals were handled and placed back into their same color environments and corticosteroid levels increased over time with no commensurate change in skin color. Changes in the distribution of melanosomes within the melanophores did not correlate with corticosteroid levels (Fig. 6C), but did correlate with plasma αMSH levels and light exposure (Fig. 5B).

## Conclusions

The color change response showed clear divisions along crocodylian phylogenetic lines (Fig. 1). While these data alone cannot be used as a metric for the determination of taxonomic relationships, they are supportive of the current accepted phylogeny (Fig. 2). Members of the genus *Crocodylus* respond relatively rapidly to ocular light stimulation, while other members of the Family Crocodylidae and all members of the Family Alligatoridae do not respond in this manner. This response most likely developed to allow the common ancestor to the members of the genus *Crocodylus* to evade predators when they are young, by blending with background color. In addition, this adaptation would have given an advantage to feeding adults. The absence of color change response by the dwarf crocodile and slender-snouted crocodile, as compared to all other extant members of the Family Crocodylidae (Fig. 2), supports the removal of the slender-snouted crocodile from the genus *Crocodylus* and the generation of the monophyletic genus *Mecistop*s^44^. In addition, the data support the potential for the slender-snouted crocodile and dwarf crocodile as an outgroup to the genus *Crocodylus* with a common ancestor^45^ (Fig. 2). Members of the Family Gavialidae exhibit a similar, but opposite effect. Gharial skin coloration darkens when exposed to light environments, and lightens in darker environments. This is likely an adaptation to aid in crypsis, similar to the countershading response described in fish^46–48^.

There has been much speculation concerning whether the Malayan gharial (*Tomistoma schlegelii*) should be placed in Family Crocodylidae or Gavialidae. While the morphological information collected from paleontological and comparative anatomical studies place the Malayan gharial within the Crocodyloidea^49,50^, the molecular data sets clearly place the it in the Family Gavialidae. The data presented in this study (Fig. 1) support the inclusion of the Malayan gharial in the Family Gavialidae, as a sister taxon to the Indian gharial^51–53^ (Fig. 2).

## Methods

### Treatment of Animals

Because the animals used in this study were housed in a broad spectrum of zoo facilities and private collections, they were maintained in tanks of various dimensions and were fed different diets. Blood was collected from the spinal vein as previously described^54^ using 23 ga. needles and 5 mL syringes. All of the methods utilized in this study were approved by the McNeese State University Animal Care and Use Committee, and when necessary, institutional ACUC protocols were approved. In addition, all of the procedures were conducted such that they were in accordance with the permissions and approvals granted by these institutions.

### Measurement of skin color

Brightness of crocodylian skin was measured using a Konica-Minolta CR-410 chromameter. The instrument was calibrated prior to every use. The instrument was held perpendicular to the skin surface, and the reflective color (L*a*b*)^55^ measured as the skin reflected the flash from a xenon bulb. During the flash, extraneous light was blocked from the skin surface with an aluminum hood. The L* term was used to measure lightness of the skin, on a gray scale from 0 (black) to 100 (white). The same person (M. Merchant) collected all of the crocodylian skin measurements for this entire study.

Different light intensities to which crocodylians were exposed was measured using a Sinotech digital illuminance meter, and expressed in lux. Animals were exposed to each intensity for two hours, at which time changes to the skin color had stabilized. Spectral filters were used to determine the effects of different wavelength ranges. The plastic filters were taped to the bulb such that different wavelength ranges were filtered. The illuminance meter was used to adjust the light intensities such that they were the same for all treatment groups.

### α-melanocyte stimulating (αMSH) hormone assay

Plasma αMSH levels were determined by competitive enzyme-linked immunoassay (Lifespan Biosciences, Inc., Seattle, WA, USA) directed toward human αMSH, according the instructions provided by the manufacturer. Crocodylian αMSH shares 100% amino acid sequence identity with the human protein.

### Crocodylian skin histology

Punch biopsies (8 mm) were collected from crocodilian skin. Biopsies were fixed in formalin, embedded in paraffin, sectioned to 7 µm, and mounted onto glass slides. The tissues were then deparaffinized and cover-slipped without staining. Slides were imaged using a Nikon Eclipse 50i microscope and DXM 1200 F digital camera.

### Statistics and controls

The results for each experiment are presented as the means ± standard deviation for the number of animals shown in each experiment.

### Data availability

The datasets generated during and/or analysed during the current study are available from the corresponding author on reasonable request. All image acquisition tools are listed in the Methods.
